# CDCP1 (CUB domain containing protein 1) is a potential urine-based biomarker in the diagnosis of low-grade urothelial carcinoma

**DOI:** 10.1371/journal.pone.0281873

**Published:** 2023-03-02

**Authors:** Chien-Liang Liu, Hung-Wen Tsai, Shu-Ling Peng, Ching-Ping Chang, Yu-Hao Chang, Huei-Sheng Huang

**Affiliations:** 1 Division of Urology, Department of Surgery, Chi Mei Medical Center, Tainan, Taiwan; 2 Department of Pathology, National Cheng Kung University Hospital, College of Medicine, National Cheng Kung University, Tainan, Taiwan; 3 Department of Medical Research, Chi Mei Medical Center, Tainan, Taiwan; 4 Department of Medical Laboratory Science and Biotechnology, College of Medicine, National Cheng Kung University, Tainan, Taiwan; Chung Shan Medical University, TAIWAN

## Abstract

Urine-based cytology is non-invasive and widely used for clinical diagnosis of urothelial carcinoma (UC), but its sensitivity is less than 40% for low-grade UC detection. As such, there is a need for new diagnostic and prognostic biomarkers of UC. CUB domain containing protein 1 (CDCP1) is a type I transmembrane glycoprotein highly expressed in various cancers. Using tissue array analysis, we demonstrated that CDCP1 expression in UC patients (n = 133), especially in those with low-grade UC, was significantly higher than in 16 normal persons. In addition, CDCP1 expression in urinary UC cells could also be detected by using immunocytochemistry method (n = 11). Furthermore, in 5637-CD cells, overexpression of CDCP1 affected the expression of epithelial mesenchymal transition-related markers and increased matrix metalloproteinase 2 expression and migration ability. Conversely, the knockdown of CDCP1 in T24 cells had the opposite effects. Using specific inhibitors, we demonstrated the involvement of c-Src/PKCδ signaling in the CDCP1-regulated migration of UC. In conclusion, our data suggest that CDCP1 contributes to the malignant progression of UC and may have the potential as a urine-based biomarker for detecting low-grade UC. However, a cohort study needs to be conducted.

## Introduction

Urothelial carcinoma (UC) of the bladder is estimated to be the 4^th^ commonly diagnosed cancer and ranked 8^th^ in cancer-related death in males in the United States in 2022 [1]. Some risk factors for the high prevalence of UC worldwide include tobacco smoking and ingestion of arsenic contaminated water or herbal medicines containing aristolochic acid. However, the exact mechanism of the disease is not yet fully elucidated.

The high recurrence rate of UC causes the need for long-term surveillance by regular cytologic and cystoscopy examinations for follow-up UC patients [2]. Presently, cystoscopy or a combination of urinary cytology is the gold standard for UC diagnosis. However, cystoscopy is unsuitable for screening UC patients because the frequently invasive operation might make patients uncomfortable and incur high medical costs. The major advantages of urinary cytology with the Papanicolaou (PAP) stain method are its non-invasiveness, lower turnaround time in the laboratory, and cost-effectiveness, and has been widely used as a screening or a follow-up tool for patients not only in UC but also in cervical cancer [3, 4]. One disadvantage of the PAP stain method is the requirement of a well-trained cytopathologist to clarify malignant cells according to their morphological features, such as a high nuclear/cytoplasmic ratio (N/C), aggregating clusters, increased cellularity, irregular nuclear margins, hyperchromasia, and chromatin abnormality [5]. Nonetheless, there are still no standard criteria for the morphological evaluation, especially in an atypical status, which often confuses experienced cytopathologists [6, 7]. Further, preserved or inadequate urine specimens, clinical conditions, and laboratory processing also influence the accuracy and quality of cytology. Therefore, urinary cytology with the PAP stain method is suitable for high-grade UC diagnosis and prognosis; however, its low sensitivity for detecting low-grade UC is a concern [8, 9]. In brief, finding new diagnostic and prognostic biomarkers of UC is eagerly needed to improve the sensitivity of urinary cytology.

CUB domain-containing protein 1 (CDCP1) is a type I transmembrane glycoprotein. Full-length CDCP1 (135–140 kDa) comprises three domains. The extracellular domain can be cleaved at the R368 and K369 by some proteases, such as matriptase, plasmin, trypsin, and urokinase, generating a smaller C-terminal transmembrane fragment (75–85 kDa) [10, 11]. The CUB (complement C1r/C1s, Uegf, and Bmp1) domain has been suggested to play an important role in the developmental process [12]. It also plays a critical role in tumor metastasis to regulate anoikis resistance in lung cancer cells [13]. It promotes pancreatic cancer migration, invasion, and extracellular matrix (ECM) degradation in a phosphorylation-dependent manner [14]. CDCP1 can mainly serve as a substrate for the binding of Src family kinase (SFK), including Src, Fyn, and Yes, at Y734 of the cytoplasmic domain to recruit PKCδ to the membrane and interact with Y762 site to induce anoikis resistance, cell migration, and extracellular matrix degradation [13]. It can also interact with other receptor tyrosine kinases and cellular surface proteins as a hub to relay signaling to modulate cancer progression [15]. Moreover, CDCP1 is overexpressed in various cancers, including colon, kidney, lung, breast, pancreas, liver, ovary, and prostate cancers [15]. However, the roles of CDCP1 in UC are not well-elucidated. Recently, it is reported that the proteolytic neoepitopes of CDCP1can be recognized by a specific antibody conjugated with a drug to enable more effective target treatments for solid tumors [16]. Evidence also reports its promising roles as a diagnostic biomarker and therapeutic target for human cancer [17].

In the present study, UC tissue microarray slide analysis indicated significantly higher CDCP1 expression in low-grade UC than in normal tissues. Its expression could also be measured in urinary UC cells of patients. In addition, CDCP1 regulated migration/invasion ability of UC cells through the c-Src/PKCδ signaling. Therefore, its potential application in the urinary cytology of UC is suggested.

## Materials and methods

### Reagents and antibodies

SuperScript™III, RPMI 1640 medium and Opti-MEM medium were obtained from Invitrogen (Carlsbad, CA). TriSolution Plus Reagent was from GeneMark (Atlanta, Georgia). RNAzol^®^ RT RNA isolation reagent was purchased from Molecular Research Center, INC (Cincinnati, OH). GoScript^TM^ Reverse Transcriptase, Go Taq^®^ Green Master Mix (2X), and luciferase assay system were from Promaga (Madison, WI). G418 disulfate salt was from Sigma-Aldrich (St. Louis, MO). Src inhibitor PP1 was from Calbiochem (Merck Millipore, Darmstadt, Germany). Src inhibitor-1 was from Merck Millipore (Burlington, MA, USA). HyFect^TM^ DNA transfection reagent was from Leadgene Biomedical (Tainan, Taiwan). Antibodies against *β*-actin and Flag were obtained from Sigma-Aldrich (St. Louis, MO). E-cadherin and N-cadherin antibodies were obtained from BD Bioscience (Bedford, MA). MMP-2 antibody was purchased from Millipore (Billerica, MA). Antibodies against CDCP1, c-Src, pSrc^Tyr416^, and pPKCδ^Tyr314^ were purchased from Cell Signaling Technology (Danvers, MA).

### Cell culture

Human UC cell line Tri-Service General Hospital-8301 (TSGH-8301) was provided by Dr. Dah-Shyong Yu (Division of Urology, Tri-Service General Hospital, National Defense Medical Center, Taipei, Taiwan). BFTC905, 5637, and T24 cells were obtained from the Bioresource Collection and Research Center (BCRC, Hsinchu, Taiwan). Cells were cultured in RPMI 1640 medium containing 10% fetal calf serum (FCS), 2 mM glutamine, 100 μg/ml streptomycin, 100 U/ml penicillin (Invitrogen, Co.) and incubated at 37°C in a 5% CO_2_ humified incubator for cell culture.

### Western blot

An analytical 10% SDS-PAGE was performed. Cell lysates prepared from each reaction were separated by SDS-PAGE, and then followed by transferring to a polyvinylidene difluoride (PVDF) membrane on a semidry apparatus. For immunoblotting, specific antibodies against target genes were employed as primary antibodies. Rabbit or mouse lgG antibody coupled with horseradish peroxidase was used as a secondary antibody. Then the protein expression was detected by an enhanced chemiluminescence kit (Amersham). The density of the immunoblots was measured by an image analysis system installed with a software BIO-ID (Vilber Lourmat, France).

### RT-PCR

The mRNA expression of targeted genes was analyzed by RT-PCR. Total RNA was isolated by using RNAzol^®^ from various cells and was reverse-transcribed into first strand cDNA according to the manufacture’s instruction of GoScript^TM^ reverse transcriptase. Specific primers were designed from each gene sequence. Primers used in the RT-PCR were described as follows: *CDCP1* primers (sense: 5’-GGGTCTGACGGTGTCCTTT-3’; antisense: 5’- CTGCCTCGGCATCTCAGTAT -3’); *mmp2* primers (sense: 5’-CAACTACGATGATGACCGCAA-3’; antisense: 5’-GTGTAAATGGGTGCCATCACG-3’); *e-cadherin* primers (sense: 5’- TCCCATCAGCTGCCCAGAAA-3’; antisense: 5’- TGACTCCTGTGTTCCTGTTA-3’); *n-cadherin* primers (sense: 5’- CATCTGGACGAAATCAGAACC-3’; antisense: 5’- CCAAATGTATGTTGAGGACTGC-3’); *gapdh* primers (sense: 5’-CCATCACCATCTTCCAGGAG-3’; antisense: 5’-CCTGCTTCACCACCTTCTTG-3’). PCR were then carried out for 25 to 35 cycles with a suitable program to amplify the gene products in a 2720 thermal cycler (Applied Biosystems, Foster City, CA). The PCR products were size-fractionated by electrophoresis in a 1.5% agarose gel, stained with 0.1 μg/ml ethidium bromide and photographed by ultraviolet light illumination. The *gapdh* gene was used as an internal control. Quantification of the results was carried out by an image analysis system installed with a software UN-SCAN-IT gel 6.1.

### Plasmid construction and lentivirus transduction

The full-length cDNAs of the human CDCP1 gene were amplified by RT-PCR from total RNA of TSGH8301 cells with 2 pairs of primers as follows: pair-1 bearing EcoRV/BamHI as linkers (sense: 5’-GATATCATCGCCGGCCTGAACTGC-3’; antisense: 5’-CTCGATGGTGAGTGACATGGCTCG-3’); and pair-2 bearing BamHI/KpnI as linkers (sense: 5’-TCTCTGCAAGGCTGTGACCAAGTGCC-3’; antisense: 5’-GGTACCTTATTCTGCTGGCTCCATGGG-3’). The two PCR products were then cloned into T&A^TM^ cloning vector (Yeastern Biotech, Taipei, Taiwan), and subcloned into the pcDNA3.1 (-) expression vector with EcoRV and KpnI digestion (pcCDCP1), or into pLenti-blast backbone vector to generate pLenti-CDCP1 plasmid. The clones were selected and validated by DNA sequencing. On the other hand, the plasmids of pLKO.1-CDCP1-shRNA#1, pLKO.1-CDCP1-shRNA#2, pLKO.1-CDCP1-shRNA#3, targeting at the human CDCP1 gene sequence 5’- CCATCAAGTCTGGAGAAAGAA-3’, 5’- CATTGCAAACCGCTCATCTAT-3’, and 5’- CGTCTCCTTCCTCAACTTCAA-3’, respectively, were purchased from National RNAi Core Facility located at the Institute of Molecular Biology/Genomic Research Center, Academia Sinica (Taipei, Taiwan). The pLKO.1-shLuc was used as a control. The transfection method for pcCDCP1 was based on the manufacturer instruction of HyFect^TM^ DNA transfection reagent with a slight modification. Cells (5×10^5^/dish) were cultured onto 60-mm dishes with normal medium for 24 hrs. Then the medium was replaced with a mixture containing plasmids and HyFect^TM^ DNA transfection reagent in 0.2 ml of Opti-MEM to incubate at RT for 30 mins, and fresh medium was added into the dishes to incubate at 37°C in a humid atmosphere of air/CO_2_ (19:1) for another 24 hrs. The transfection efficiency was measured by RT-PCR or Western blot. For lentivirus transduction, pLenti-CDCP1 was transduced into 5637 cells, and pLKO.1-shLuc and pLKO.1-CDCP1-shRNAs were transduced into T24 cells by lentivirus, respectively. After 18 hrs transduction, these UC cells were selected by 10 μg/ml blasticidin or 5 μg/ml puromycin for further 14 days to obtain stable transfectants, and designated 5637-CD, shluc, shCD#2, and shCD#3 cells.

### IHC for tissue microarray slides and ICC for urinary cytology

For IHC of CDCP1, paraffin-embedded human bladder tissue microarray slides were purchased from Biomax (BLC1501, BL601a, and BL208, Biomax Inc., Rockville, MD, USA). The patients’ clinical information including gender, age, stage, grade, TNM, and type was provided in S1 Table. These slides were deparaffinized with 100% xylene for 3 mins 3 times, rehydrated with 95% ethanol for 5 mins 2 times, 75% ethanol for 5 mins twice, 50% ethanol for 2 mins, 30% ethanol for 2 mins, and washed with 1x PBS for 5 mins twice. For ICC of CDCP1, residual urine samples were used. The study was performed according to the Helsinki declaration and was approved by the institutional review board of NCKUH (No. B-ER-104-036). The institutional review boards waved the need for written informed consent due to the process involved no potential risk to patients. The urine specimens of UC patients (n = 11) including voided urines and instrumented urines (bladder/ureteral wash and postcystoscopic urine) were validated by experienced cytopathologist of NCKUH (Department of Pathology). The evaluation of cancerous status followed the criteria outlined by The Paris System for Reporting Urinary Cytology (TPS), such as high N/C ratio and eccentric, enlarged, and hyperchromatic nuclei [5]. Eleven urine specimens were concentrated by a Cytospin slide centrifuge at 1500 rpm for 5 mins for urinary ICC detection. Cells were fixed on the slide with 95% ethanol for 30 mins. Both of the tissue microarray slides and urine specimens were oxidized by using 3% H_2_O_2_/methanol for 10 mins and washed with 1x PBS for 5 mins twice. The antigen retrieval was performed in 0.01 M citrate buffer (pH 6.0) by microwave heating for 30 mins. After cooling down, the slides were washed with 1x PBS for 5 mins 2 times and drawn a hydrophobic circle by a Dako PAP pen. The samples were blocked by CAS-Block buffer for 1 h, followed by incubation with monoclonal antibody against CDCP1 (1:100; LifeSpan BioSciences, Seattle, WA) at 4°C for 18 hrs. After washing with 1x PBS for 5 mins twice, the samples were incubated with secondary antibody (Invitrogen, Frederick, MD) for 7 mins, stained with diaminobenzidine (DAB) for 5 mins, and then added hematoxylin (MUTO Pure Chemicals, Tokyo, Japan) for counterstain.

### Cellular migration/invasion assay

Transwell^®^ chamber with 8 μm pore size (Corning, Corning, NY) was used for the cellular migration/invasion assay. After transfection with various plasmids for 48 hrs, cells were harvested and resuspended in serum-free medium, then the cells (1.5 x 10^4^) were added to the upper chamber with uncoated polycarbonate membrane for migration assay, or with matrigel-coated (BD Bioscience, Bedford, MA) membrane for invasion assay, respectively. RPMI1640 medium supplemented with 10% FBS were placed into each well of the bottom chamber to act as a chemoattractant. After incubation for 12 hrs at 37°C, cells on the upper side of membrane were removed by a cotton swab. The migrating cells to the bottom surface of the membrane were fixed with 100% methanol for 10 mins, stained with 10% Giemsa for 30 mins, and counted under a microscope in 5 random fields (100 X) per well and then quantified by a software Image-J. Values are means±SD for three determinations.

### Colony formation in soft agar

Anchorage-independent cell growth was determined by analyzing the formation of colonies in soft agar. 6-well plates were precoated with 0.6% agarose/RPMI-1640 with 10% FBS, and the cells were seeded at 6×10^3^ cells per well in 0.3% agarose/RPMI-1640 with 10% FBS. The cells were incubated for 2 weeks and then stained with 0.5% crystal violet solution and washed with water extensively to remove excess stain. Colonies with a diameter greater than 1 mm were counted under an inverted microscopic field at 40Χmagnifications. Means were based on numbers from triplicate wells for each treatment condition and were analyzed using two-sided Student’s t test.

### Statistical analysis

All experiments were performed for at least three times. A representative result was presented. Differences between the bladder tissue groups were statistically evaluated by one-way ANOVA with Tukey’s multiple comparisons test, which was performed by using GraphPad Prism 7.01 software. Statistical analyses of cellular experiments were evaluated by unpaired two-tailed Student’s t-test. The results were presented as mean ± SD (n = 3, *p < 0.05, **p < 0.01, ***p < 0.001).

## Results

### Increased CUB domain containing protein 1 expression is associated with histologic grades of urothelial carcinoma patients

**Fig 1:**
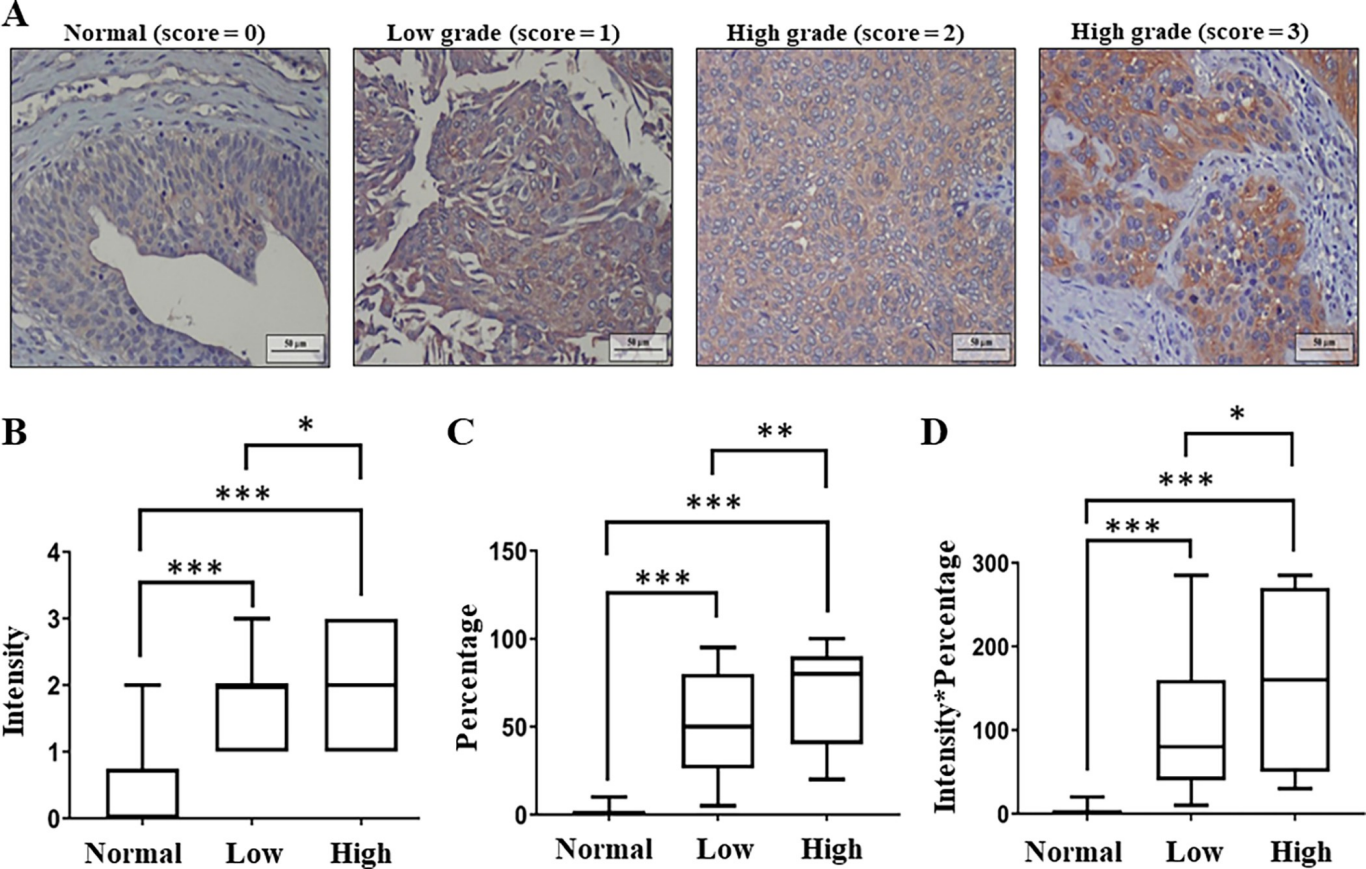
Evaluation of the clinical significance of CDCP1 by using tissue arrays. (A) Immunohistochemical staining with antibody against CDCP1 was performed to evaluate CDCP1 expression on the tissue arrays of UC. IHC tissues were observed and photographed at 40X magnification with an optical microscope (Olympus, Tokyo, Japan). Images were representative of negative, weak, moderate and strong CDCP1 staining at specimens of the normal, low grade, and high grade. (B) The intensity of CDCP1 expression was correlated with tumor grade. (C) The staining percentage of CDCP1 was correlated with tumor grade. (D) The product of the intensity and staining percentage of CDCP1 was correlated with tumor grade. Scale bar indicates 50 μm. Statistical analyses were evaluated by one-way ANOVA with Tukey’s multiple comparisons test. The results were presented as mean ± SD (normal n = 16; low grade n = 76; high grade n = 57, *p < 0.05, **p < 0.01, ***p < 0.001).

According to the analysis of the ONCOMINE database (http://www.oncomine.org), the microarray results show significantly increased CDCP1 expression in UC compared with the normal tissues [18]. To further validate the clinical significance of CDCP1 in UC, tissue arrays were evaluated using IHC staining according to the criteria as follows. The IHC intensity score ranged from 0 to 3, and the staining percentage from 0 to 100%. The images were representative of negative (IHC intensity score = 0), weak (IHC intensity score = 1), moderate (IHC intensity score = 2), and strong (IHC intensity score = 3) CDCP1 staining in normal (n = 16), low-grade (n = 76), and high-grade specimens (n = 57) (Fig 1A). Data were statistically analyzed using a student’s t-test with Prism V5.01 software (GraphPad, San Diego, CA). The intensity (Fig 1B), staining percentage (Fig 1C), and product of the intensity and percentage of CDCP1 were all associated with tumor grade with high statistical significance (Fig 1D). Furthermore, the product of the intensity and staining percentage of CDCP1 was also higher in all UC specimens (both low-grade and high-grade specimens) than in normal tissues (S1 Fig). The results indicate the potential utility of CDCP1 as a useful biomarker to differentiate tumor and normal tissue in the diagnosis using tissue specimens of UC patients. Noteworthily, the intensity of CDCP1 in low-grade UC was higher than in normal urothelium (Fig 1B), implying that CDCP1 expression might enhance the differentiation of low-grade UC in urine cytology diagnosis.

### CUB domain containing protein 1 expression can be diagnosed in patients with urothelial carcinoma by using immunocytochemistry

**Fig 2:**
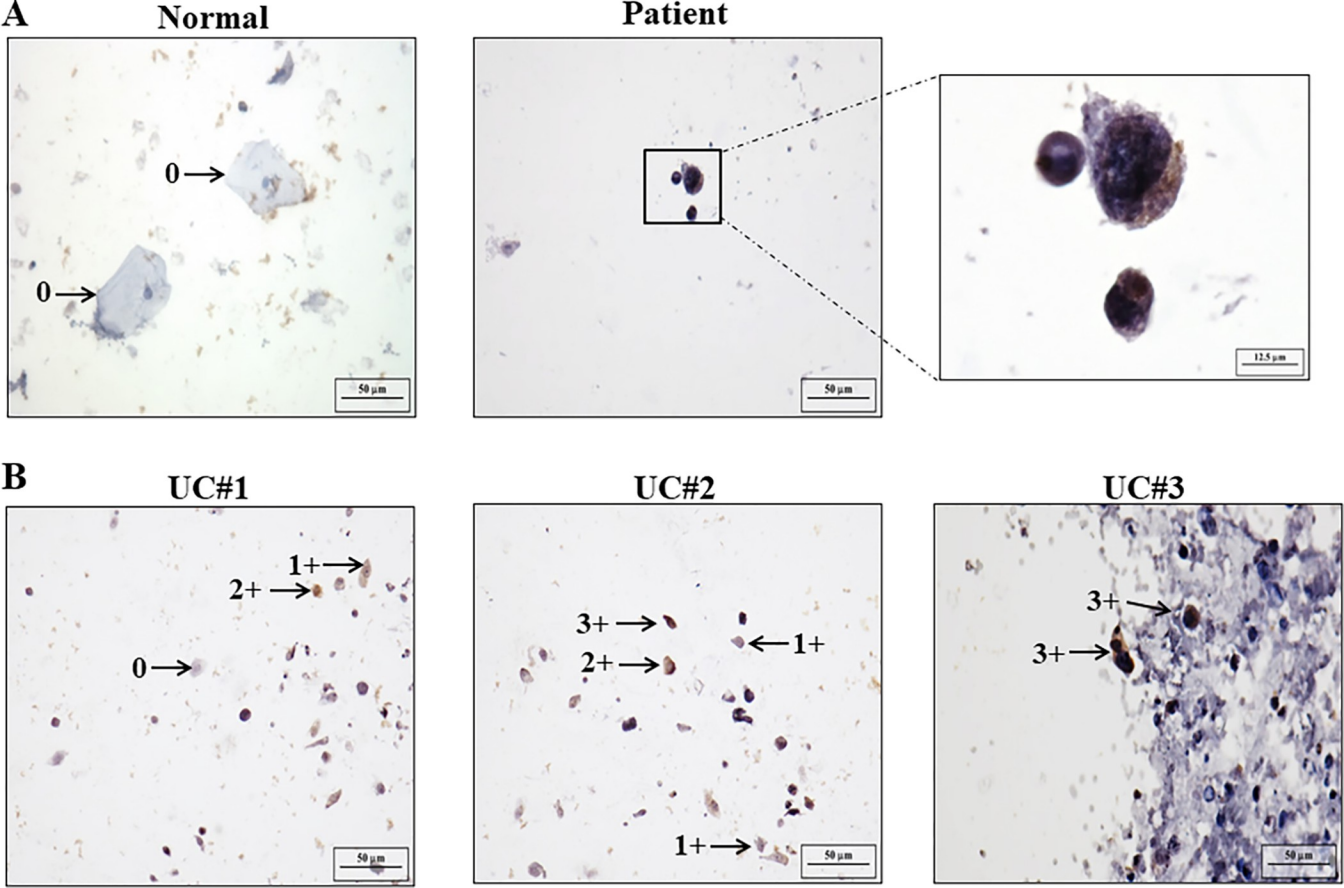
Detection of CDCP1 expression in UC patients by using urinary ICC method. Urine-based cells of UC patients were collected from NCKUH. CDCP1 ICC was performed by using its specific antibody as described in “Material and methods”. (A) Increased CDCP1 was observed in UC specimen. (B) The CDCP1 ICC images of three UC patients were presented. The criteria to evaluate CDCP1 expression were indicated as negative (ICC intensity = 0), weak (ICC intensity = 1), moderate (ICC intensity = 2) and strong (ICC intensity = 3). Images of tissue specimen and urine cytology were taken at 400x magnification. Scale bar indicates 50 μm.

Next, we got 11 urine specimens of UC patients from NCKUH, which were validated by well-trained cytopathologist according to TPS analysis. The CDCP1 ICC method was further conducted to evaluate these urine specimens. As shown in Fig 2A, increased CDCP1 was observed in UC specimen. Furthermore, the evaluation criteria were determined as follows. The ICC images of CDCP1 were representative of different valences between normal, atypical, and malignant cells from zero to three, and designated as negative (ICC intensity = 0), weak (ICC intensity = 1), moderate (ICC intensity = 2) and strong (ICC intensity = 3) CDCP1 reactivity in urine specimens of three UC patients (Fig 2B). Based on our evaluation criteria, the results indicated that the patient whose specimen contained at least five trivalent cells might have a high probability of suffering from UC. However, a cohort of patients with UC needs to be collected and studied to evaluate the applications of CDCP1 ICC in the urinary cytology of UC.

### CUB domain containing protein 1 highly expresses in muscle-invasive UC cell line T24

**Fig 3:**
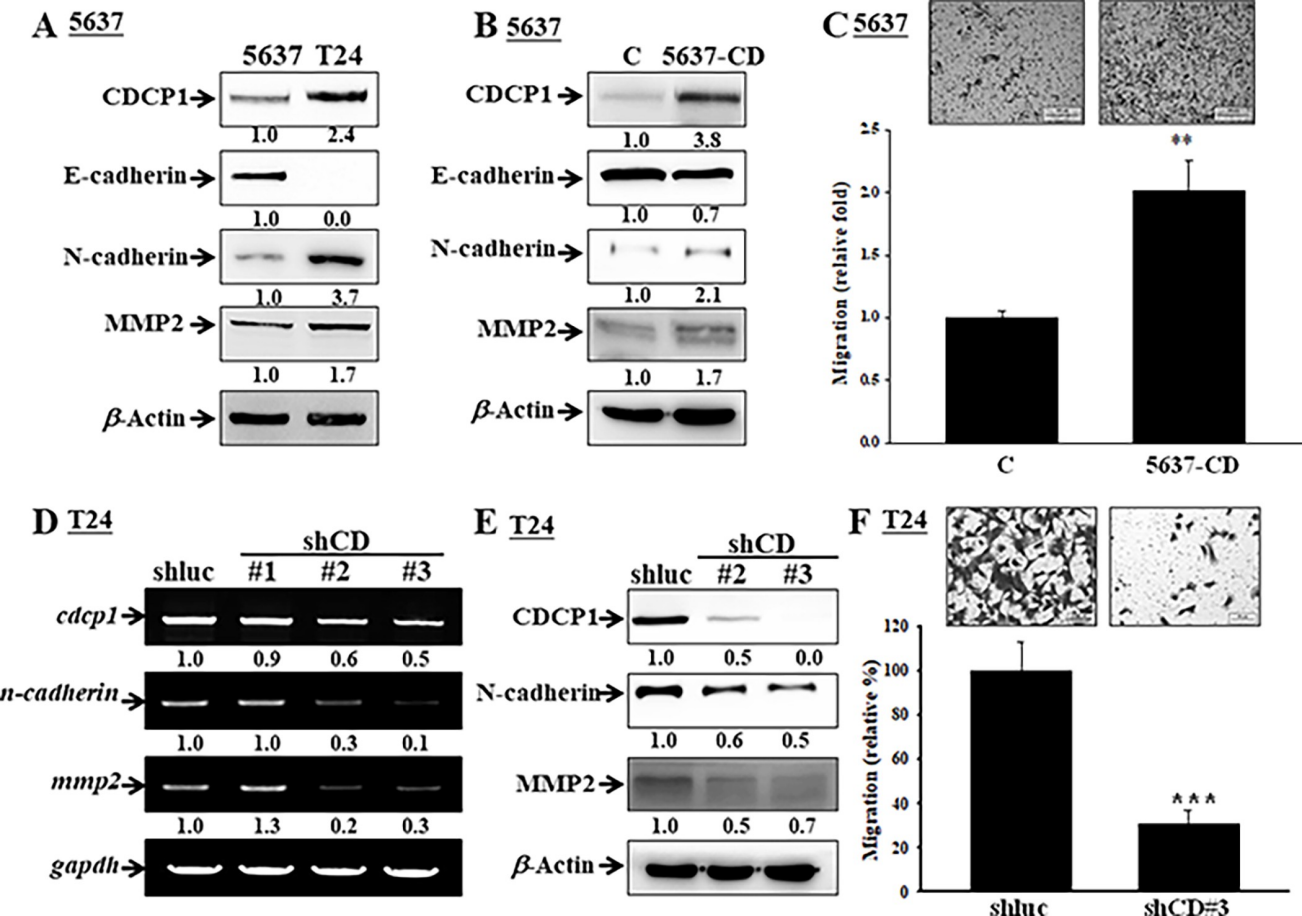
Effects of CDCP1 on the EMT markers alternation and cellular migration abilities in UC cells. Cell lysates were collected from (A) 5637 and T24 cells or from (B) parental and 5637-CD cells to perform Western blot for detecting CDCP1, n-cadherin, e-cadherin, MMP2, and *β*-actin. (C) Parental and 5637-CD cells were harvested to seed on the upper chamber of Transwell^®^, then the abilities of migration were measured as described in the “Materials and methods” section. Images of migration were taken at 200x magnification. Scale bar indicates 100 μm. In addition, T24 cells were transfected the CDCP1 specific shRNA plasmids for 24 hrs, and then harvested for the detection of (D) mRNA level, or (E) protein level of EMT markers, respectively. (F) Besides, the migration abilities of CDCP1-silenced T24 cells and parental cells were measured as described in “Materials and methods” section. Images of migration were captured at 400x magnification. Scale bar indicates 50 μm. Statistical analysis were analyzed by unpaired two-tailed Student’s t-test. The results were presented as mean ± SD (n = 3, *p < 0.05, **p < 0.01, ***p < 0.001).

Furthermore, we explored the roles of CDCP1 in the malignant progression of UC cells. The non-muscle-invasive UC cell line 5637 (grade II) and the muscle-invasive UC cell line T24 (grade III) were used in the subsequent studies. As shown in Fig 3A, highly invasive T24 cells expressed decreased E-cadherin and increased N-cadherin, matrix metalloproteinase (MMP2), and CDCP1 in protein levels than 5637 cells did (Fig 3A). It indicates that T24 cells exhibited increased migration/invasion abilities compared to 5637 cells. Importantly, these results imply that higher CDCP1 expression might be associated with the higher invasiveness of UC cells, consistent with the results of tissue arrays (Fig 1). Hence, the mode of action of CDCP1 in the migration/invasion of UC was further explored.

### Stable expression of CUB domain containing protein 1 in the 5637 cells show higher cellular migration abilities

Additionally, the pcCDCP1 plasmids were constructed and stably expressed in 5637 cells (5637-CD). As shown in Fig 3, T24 cells and 5637-CD cells expressed an increase of CDCP1, N-cadherin, and MMP2 but decreased E-cadherin expression (Fig 3A and 3B). Using cellular migration/invasion assay, 5637-CD cells significantly showed higher cellular migration abilities than parental cells (Fig 3C).

### Knockdown of CUB domain containing protein 1 in T24 cells inhibits cellular migration/invasion abilities and colony formation

Furthermore, we silenced CDCP1 using its specific shRNA in T24 cells to evaluate its migration/invasion abilities. We designed three putative shRNAs targeting the human CDCP1 gene. To evaluate off-target effects [19], we transfected these specific shRNAs in T24 cells, respectively, and observed that all three shRNA targets of CDCP1 could suppress both mRNA and protein expression of CDCP1, N-cadherin, and MMP2 (Fig 3D and 3E). Therefore, we suggested that the three shRNAs could knock down the CDCP1 expression and demonstrated the association of CDCP1 with epithelial mesenchymal transition (EMT). Accordingly, the possibility of off-target effects was limited. Given that target 3 (shCD#3) had the most effective suppression, it was selected to measure its effects on cellular migration/invasion abilities and colony formation. The results show that the knockdown of CDCP1 reduced the abilities of migration (Fig 3F) and invasion (S2A Fig), and colony formation of T24 cells (S2B Fig).

### Involvement of c-Src/PKCδ signaling in the CUB domain containing protein 1-regulated cellular migration/invasion abilities

**Fig 4:**
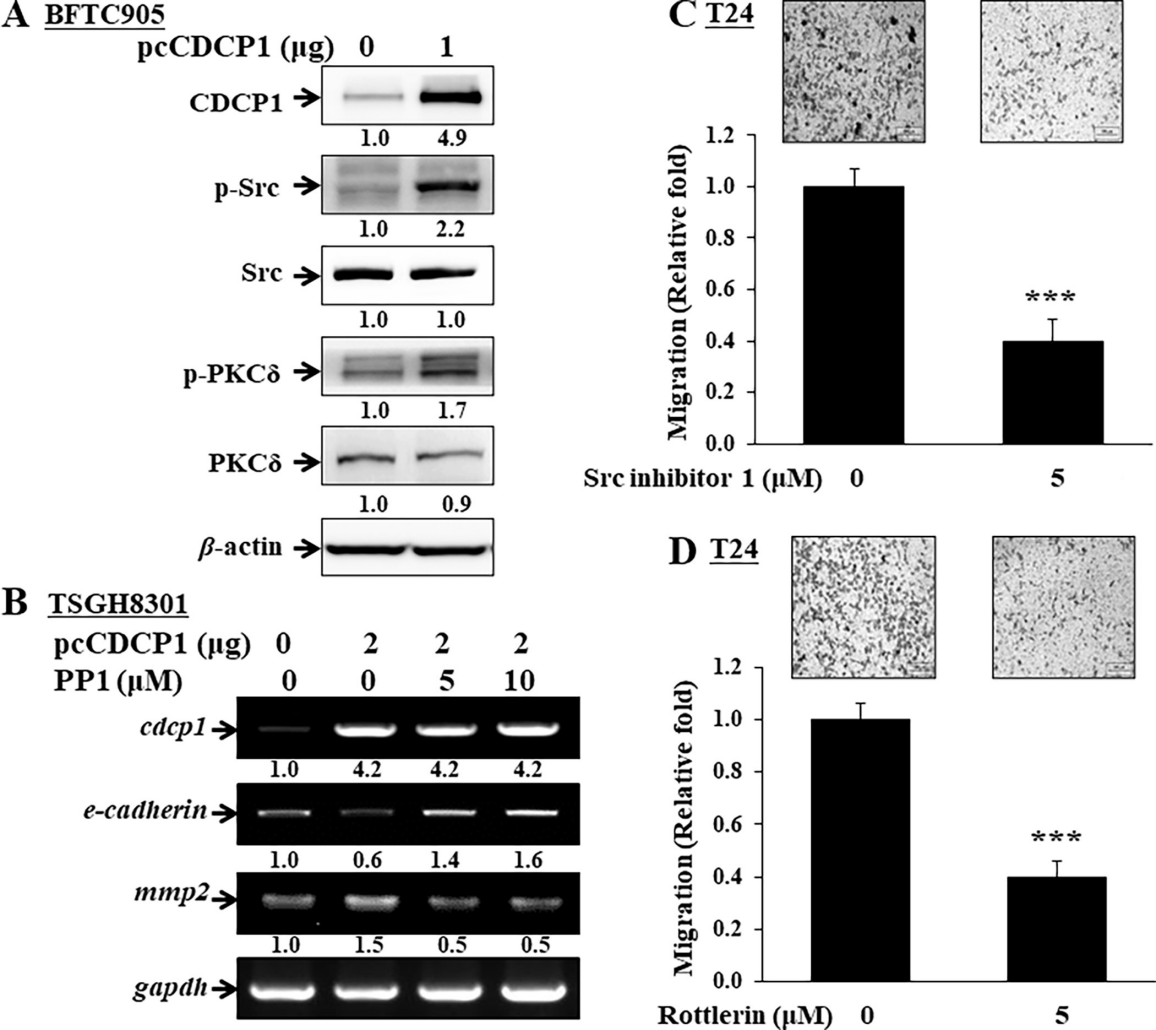
Involvement of c-Src/ PKCδ signalings in the CDCP1-regulated cellular migration abilities in UC cells. (A) BFTC905 cells were transfected with pcCDCP1 plasmids, and then harvested for the detection of phosphorylation of c-Src^Y416^ and PKCδ^Tyr311^, respectively. (B) TSGH8301 cells were transfected with pcCDCP1 plasmids or with vector control for 24 hrs, then treated with specific Src inhibitor PP1 and harvested for the detection of *mmp2* and *e-cadherin* mRNA expression. T24 cells were performed and treated with (C) specific c-Src inhibitor-1, or (D) PKCδ inhibitor rottlerin, respectively. Cellular migration abilities were measured as described in the “Materials and methods” section. Images of migration were taken at 200x magnification. Scale bar indicates 100 μm. Statistical analyses were evaluated by unpaired two-tailed Student’s t-test. The results were presented as mean ± SD (n = 3, *p < 0.05, **p < 0.01, ***p < 0.001).

CDCP1 has been reported to regulate metastasis through c-Src-kinase and PKCδ signaling in various cancers [20]. However, its regulation in UC is unclear by far. According to the ONCOMINE database, the expression of c-Src kinase and PKCδ in patients with superficial bladder cancer is about 5.5- and 6.6-fold, respectively, to that in normal tissues [18]. Accordingly, overexpression of pcCDCP1 in BFTC905 cells enhanced the phosphorylation of c-Src^Y416^ and PKCδ^Tyr311^, respectively (Fig 4A). In addition, treatment with Src kinase inhibitor PP1 in CDCP1-overexpressed TSGH8301 cells attenuated MMP2 mRNA expression and increased E-cadherin mRNA expression (Fig 4B). On the other hand, the migration abilities of T24 cells were inhibited by Src inhibitor-1 (Fig 4C) and specific PKCδ inhibitor rottlerin (Fig 4D), respectively. We demonstrated that c-Src/PKCδ activation is important to the CDCP1 downstream signaling to regulate migration/invasion abilities in UC cells.

## Discussion

Even though urinary cytology is non-invasive, relatively cheap, and can reach a high specificity of up to 98% in high-grade UC diagnosis, its overall low sensitivity (less than 40%) still poses a concern when using it as a diagnostic tool, especially in the detection of low-grade UC [21]. Thus, finding good biomarkers for urine cytology diagnosis and prognosis of UC patients is an urgent issue. The novel findings in the study are summarized as follows.

First, increased CDCP1 expression in UC tissues was associated with tumor grade (Fig 1). In addition, the expression of CDCP1 in UC tissues was significantly higher than in the normal urothelium (S1 Fig). UC cells in urine express different features from normal urothelium in urinary cytology with the PAP stain method. The specimens usually show an atypical or suspicious category and cannot be easily differentiated [5]. Further, urine specimen preservation, patients with hematuria, low numbers of cells, inflammation, and bacterial infections may also interfere with the diagnosis [9]. CDCP1 is a transmembrane glycoprotein. It is reasonable to presume that its extracellular domain can be detected by a specific antibody in the urinary cytology [15]. Using urinary ICC of CDCP1, the UC specimens (n = 11) could be differentiated from normal urothelium (Fig 2). We suggest CDCP1 might be a promising diagnostic marker to improve the low sensitivity of low-grade UC detection in urinary cytology according to its obvious differentiation between low-grade UC and normal tissues (Fig 1). However, the hypothesis still needs to be demonstrated via studying a cohort of patients with UC to assess the positive and negative predictive values to evaluate the sensitivity and specificity of the urinary ICC method of CDCP1.

Second, CDCP1 contributed to the malignant progression of UC. Not only did we prove the association between CDCP1 with the tumor grade in UC tissue assay (Fig 1), but also elucidated its involvement in the migration/invasion abilities, MMP secretion, and colony formation of UC cells (Fig 3). These events are related to the malignant progression of cancer [10]. CDCP1 was also identified as a transmembrane glycoprotein that plays a role in migration/invasion events of various cancers, including colorectal, pancreatic, ovarian, gastric, breast, prostate, melanoma, renal cell carcinoma, and lung cancers [15]. However, the functional roles of CDCP1 in UC have yet to be demonstrated systemically. Our results are the first findings related to UC by far. CDCP1 is also proven to drive fatty acid oxidation and oxidative phosphorylation to promote metastasis of triple-negative breast cancer [22]. Metabolic reprogramming has been observed in the progression of several cancers, including UC [23–25]; and thus, it should be further validated in UC in the future.

Third, c-Src/PKCδ activation was involved in CDCP1-regulated cellular migration/invasion. *In vitro* results show that cleaved CDCP1 (70 kDa) can recruit SFK and interact with the SH3 domain to relay the c-Src/PKCδ signaling [13, 15]. SFKs are well-known oncogenes in various cancers that regulate tumor progression [26]. An analysis from the ONCOMINE database indicated that c-Src expression in superficial bladder cancer is about 5.5-fold to that in normal tissues [18]. In addition, the activity of c-Src is increased in low-grade human UC over the normal bladder mucosa [27, 28]. Our results also demonstrate significantly higher CDCP1 expression in low-grade UC than in normal urothelium (Fig 1B). It is reasonable to assume that CDCP1-induced malignant progression via c-Src/PKCδ signaling. We further validated the hypothesis according to the suppression of the EMT alteration and migration of UC cells using Src inhibitor PP1 and Src inhibitor-1, respectively (Fig 4).

PKCδ can be phosphorylated by CDCP1/SFK complex through the interaction of the C2 domain of PKCδ to enhance anoikis resistance and migration/invasion abilities of cancer cells [13, 14, 29]. PKCδ belongs to the PKC family of serine/threonine protein kinases with structural homology, which can regulate cell proliferation or apoptosis in various isoforms [30]. However, whether PKCδ plays the role of tumor promoter or tumor suppressor kinase in cancer is still controversial [31]; it might depend on various cancer types, such as being a suppressor in breast cancer [32], or a promoter in lung cancer [33]. Thus, targeting PKCs for cancer therapeutics is a challenge [34]. In UC cells, the migration ability of T24 cells could be attenuated using specific PKCδ inhibitor rottlerin (Fig 4D). We also provided evidence of the critical role of PKCδ activation in the malignant progression of UC.

Recently, IHC staining formalin-fixed and paraffin-embedded triple-negative breast cancers demonstrated that the expression of phosphorylated SFK at Y416 is closely associated with phosphorylated CDCP1 and PKCδ [35]. Although we suggest that c-Src activation is involved in the CDCP1-regulated UC progression, c-Src also plays a critical role in normal physiology. Therefore, it might distort the validity of the diagnosis of UC if the phosphorylated CDCP1 was used in the staining of c-Src-expressed normal tissues. Thus, a combination of biomarkers might also be considered to increase their diagnostic efficacy in UC.

**Fig 5:**
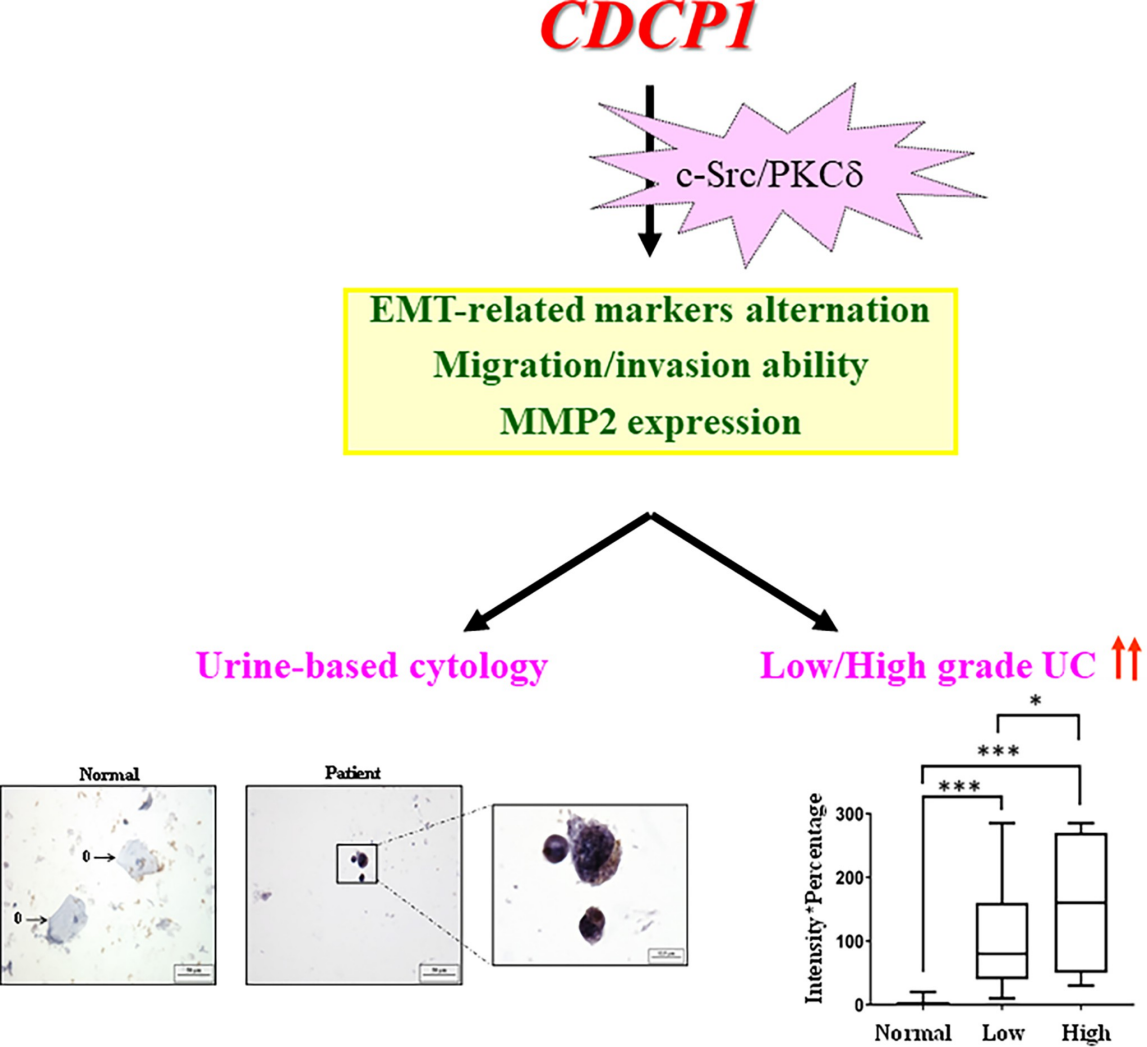
Scheme illustrates the CDCP1 as a promising cytological biomarker for detection of low-grade UC. Urine-based cytology is non-invasive and widely used for clinical diagnosis of UC, but its sensitivity is less than 40% for low-grade UC detection. We found a new biomarker CDCP1, which plays a critical role in the progression of UC and significantly increases in low-grade UC. We suggest CDCP1 may have potential as a urine-based biomarker for detecting low-grade UC in urinary cytology.

Taken together, we conclude that CDCP1 is involved in the malignant progression of UC and might be employed as a urine-based biomarker for detecting low-grade UC to fill the gaps in urinary cytology (Fig 5). Nonetheless, the UC specimens used in urinary cytology are limited and a larger scale study is needed to support our findings.

## Supporting information

S1 Raw images (PDF)

S1 Table Clinicopathologic characteristics of 133 UC patients and 16 normal persons used for IHC staining. (XLSX)

S1 Fig Evaluation of the clinical significance of CDCP1 by using tissue arrays. The product of the intensity and staining percentage of CDCP1 in specimens of UC patients was higher than that in specimens of normal persons. Statistical analyses were evaluated by two-tailed Student’s t-test. The results were presented as mean ± SD (normal n = 16; UC n = 133, *p < 0.05, **p < 0.01, ***p < 0.001).
(TIF)

S2 Fig Effects of CDCP1 knockdown in T24 cells on cellular invasion abilities and colony formation. T24 cells were transfected the CDCP1 specific shRNA plasmids for 24 hrs, (A) The invasion abilities of CDCP1-silenced cells and parental cells were measured. (B) Colony formation in soft agar was also performed to compare parental and CDCP1-silenced cells as described in “Materials and methods” section. Statistical analyses were analyzed by unpaired two-tailed Student’s t-test. The results were presented as mean ± SD (n = 3, *p < 0.05, **p < 0.01, ***p < 0.001).
(TIF)

## Abbreviations

UC: Urothelial carcinoma
CDCP1: CUB domain-containing protein 1
IHC: Immunohistochemistry
ICC: Immunocytochemistry
SFK: Src family kinase
RTKs: Receptor tyrosine kinases
FCS: Fetal calf serum
PVDF: Polyvinylidene difluoride
MMPs: Metalloproteases
N/C: Nuclear/Cytoplasmic ratio
