# Supplementary material for: CDCP1 (CUB domain containing protein 1) is a potential urine-based biomarker in the diagnosis of low-grade urothelial carcinoma
Source: PLoS One. 2023 Mar 2;18(3):e0281873. doi: 10.1371/journal.pone.0281873 (PMC9980759; doi:10.1371/journal.pone.0281873)

Fig. 3A anti-CDCP1

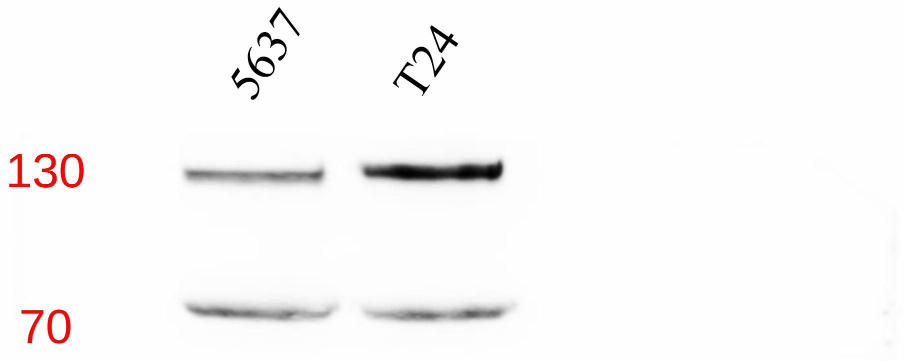

Fig. 3A anti-E-cadherin

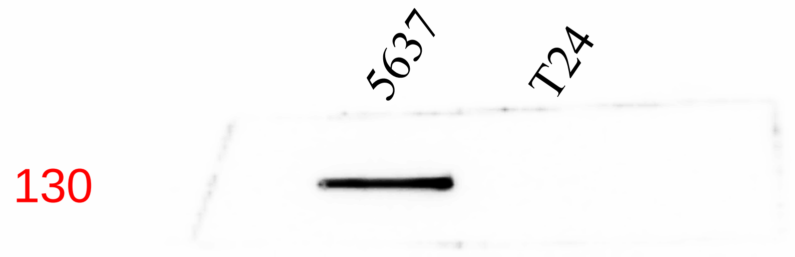

Fig. 3A anti-N-cadherin

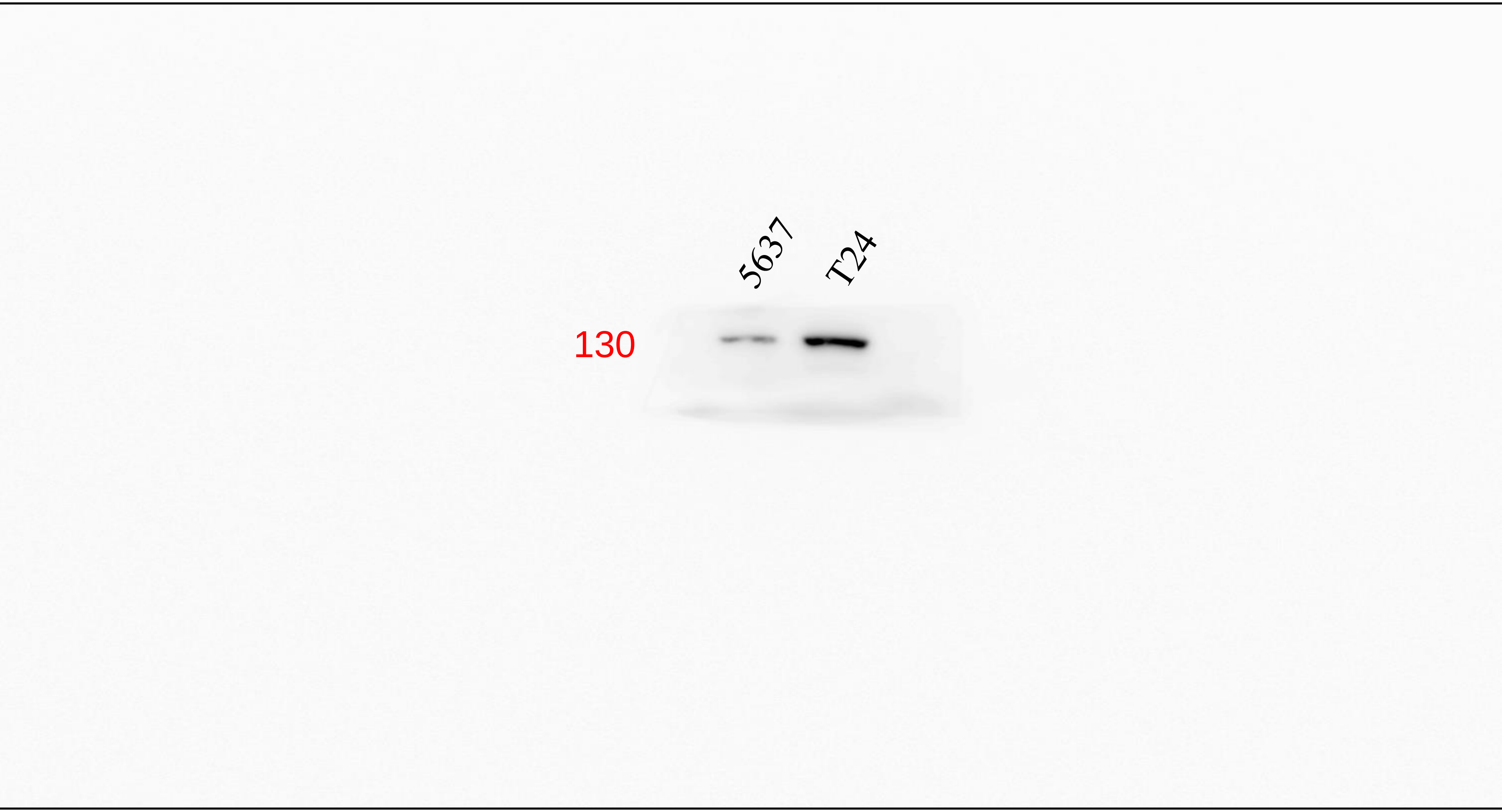

Fig. 3A anti-MMP2

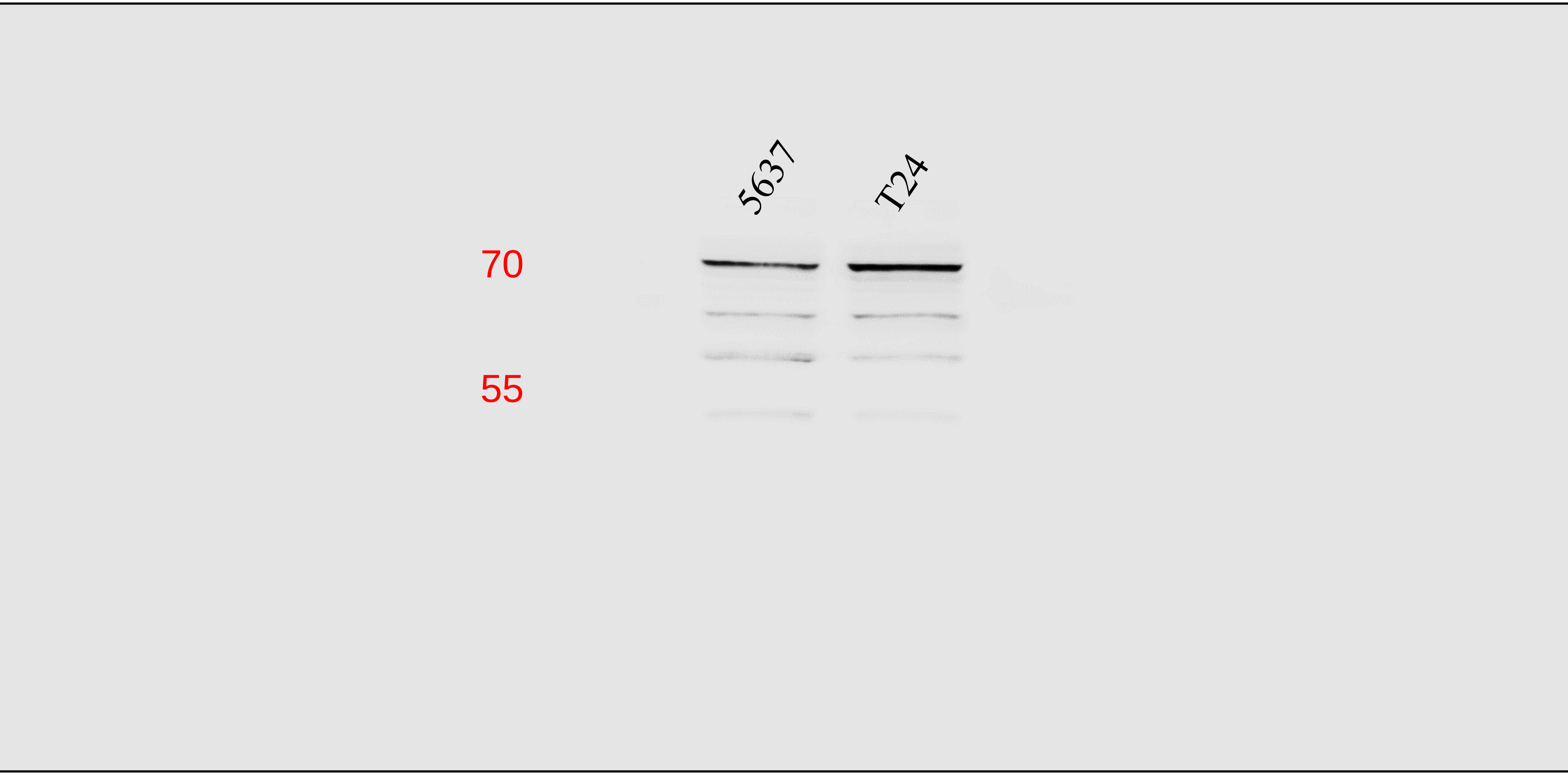

Fig. 3A anti-β-actin

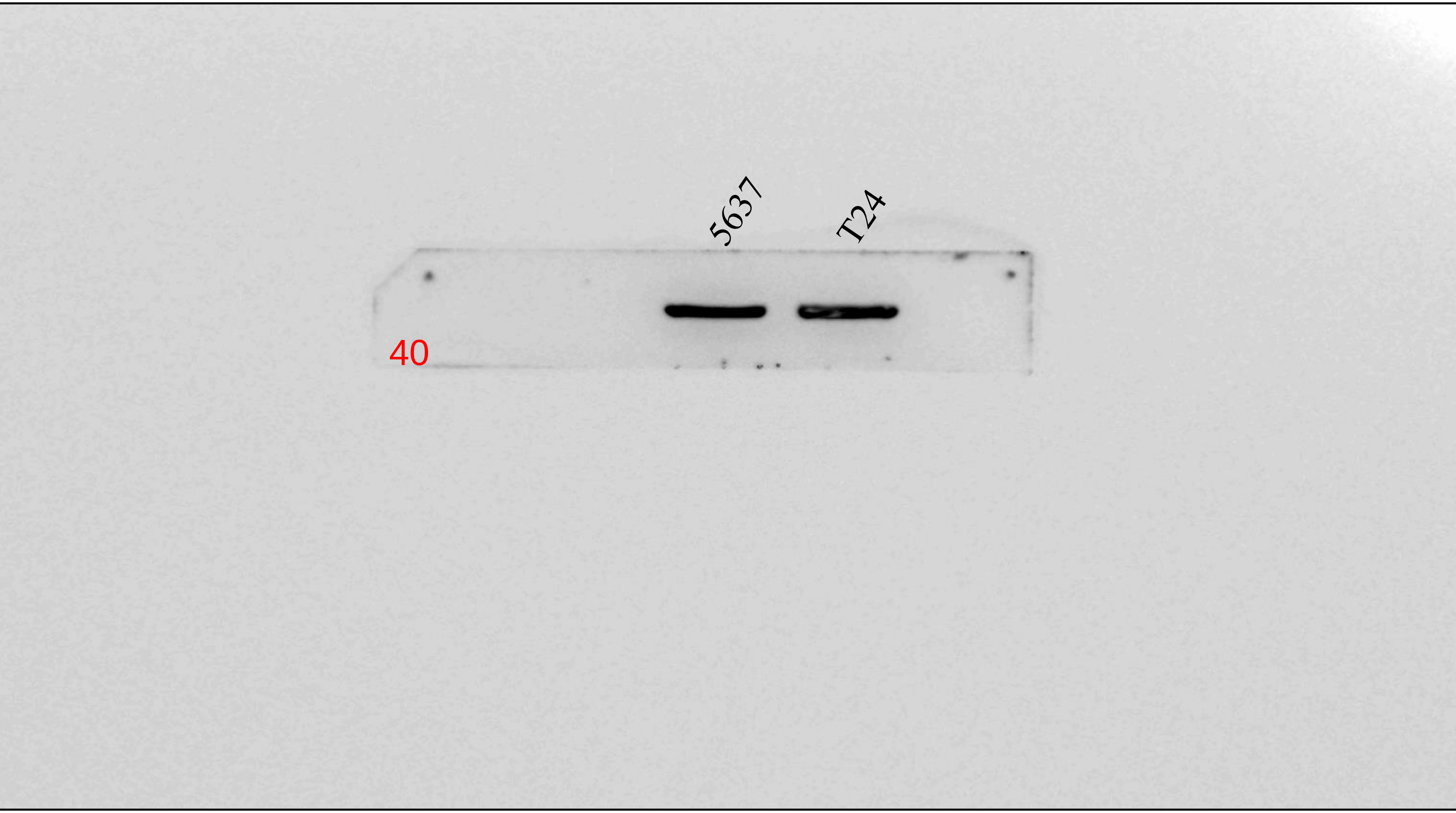

Fig. 3B anti-CDCP1

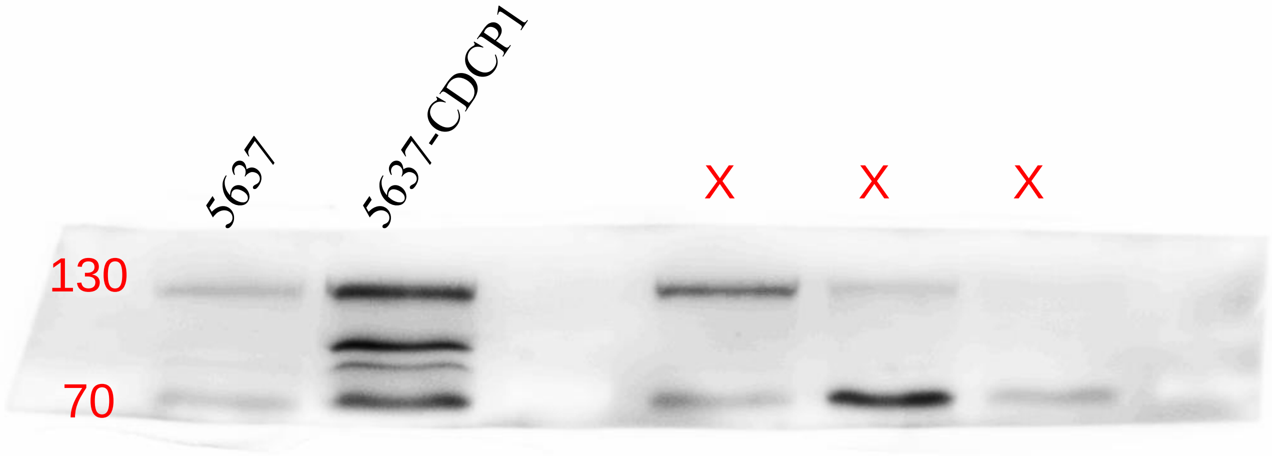

Fig. 3B anti-E-cadherin

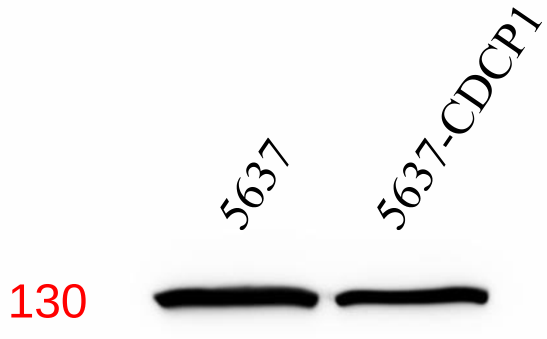

Fig. 3B anti-N-cadherin

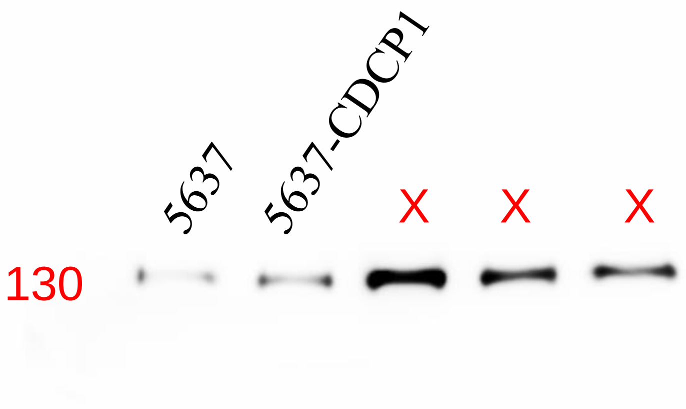

Fig. 3B anti-MMP2

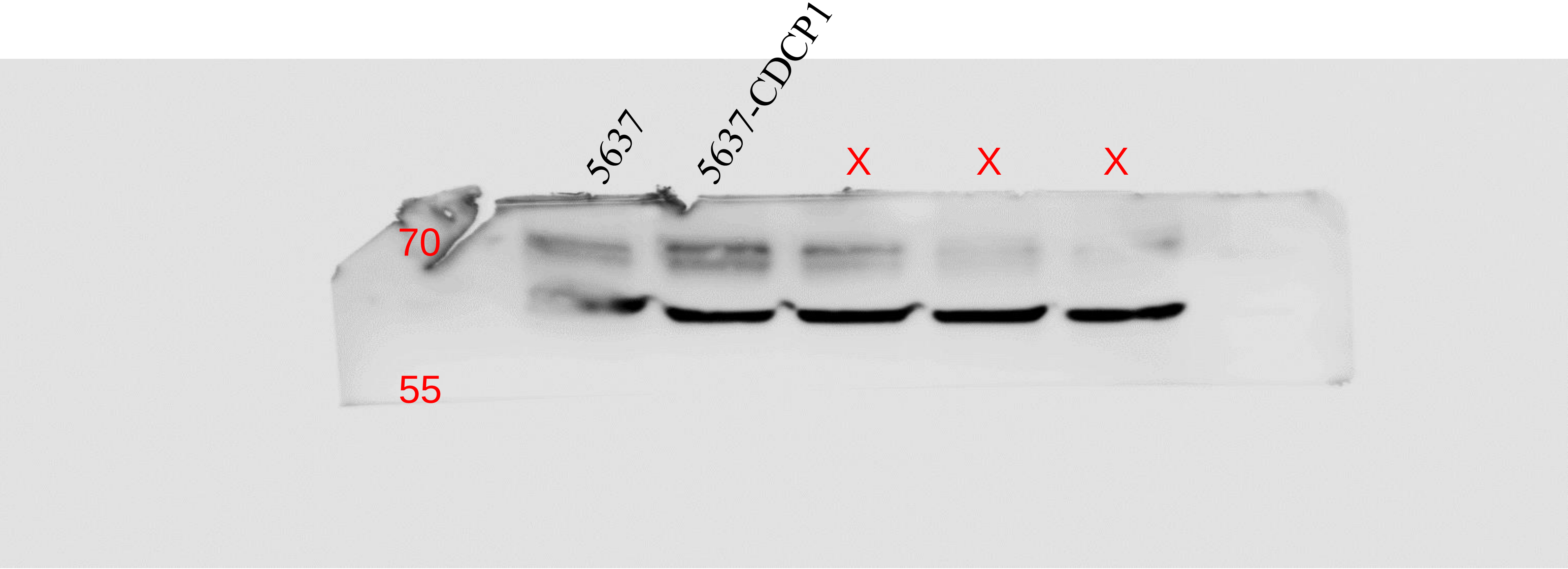

Fig. 3B anti-β-actin

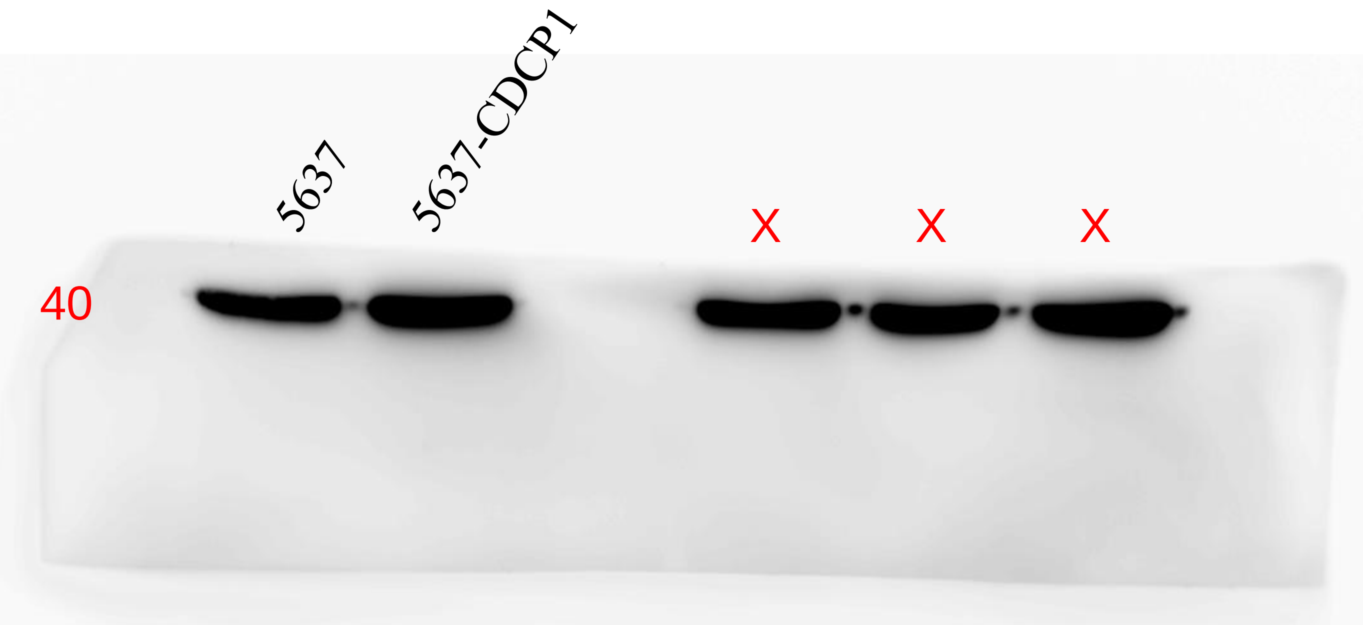

Fig. 3E anti-CDCP1

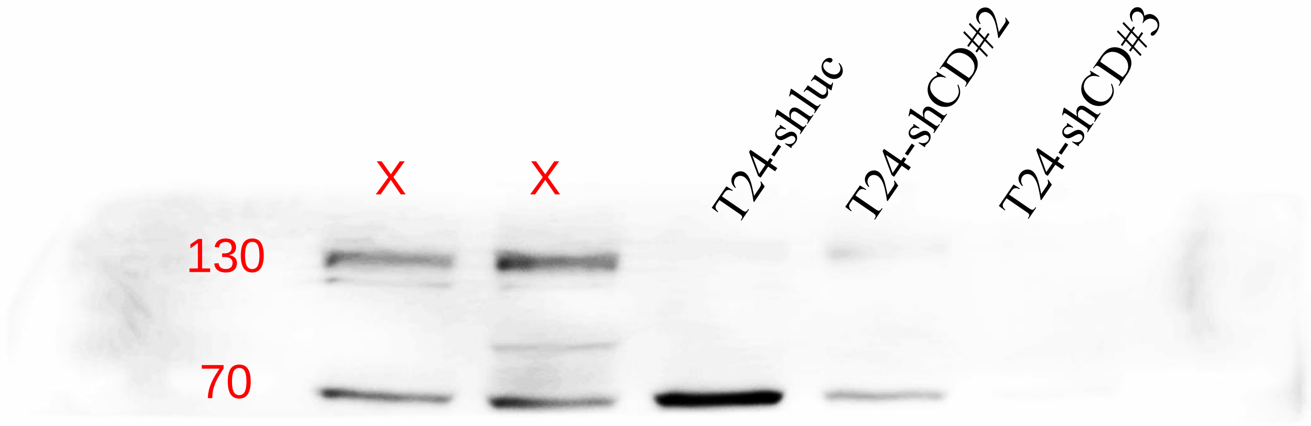

Fig. 3E anti-N-cadherin

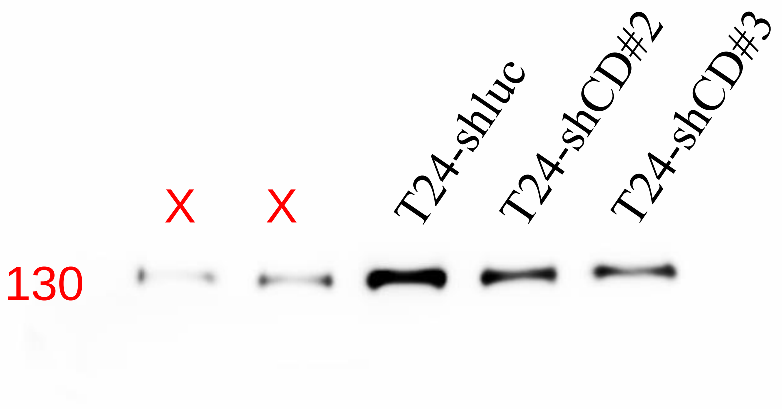

Fig. 3E anti-MMP2

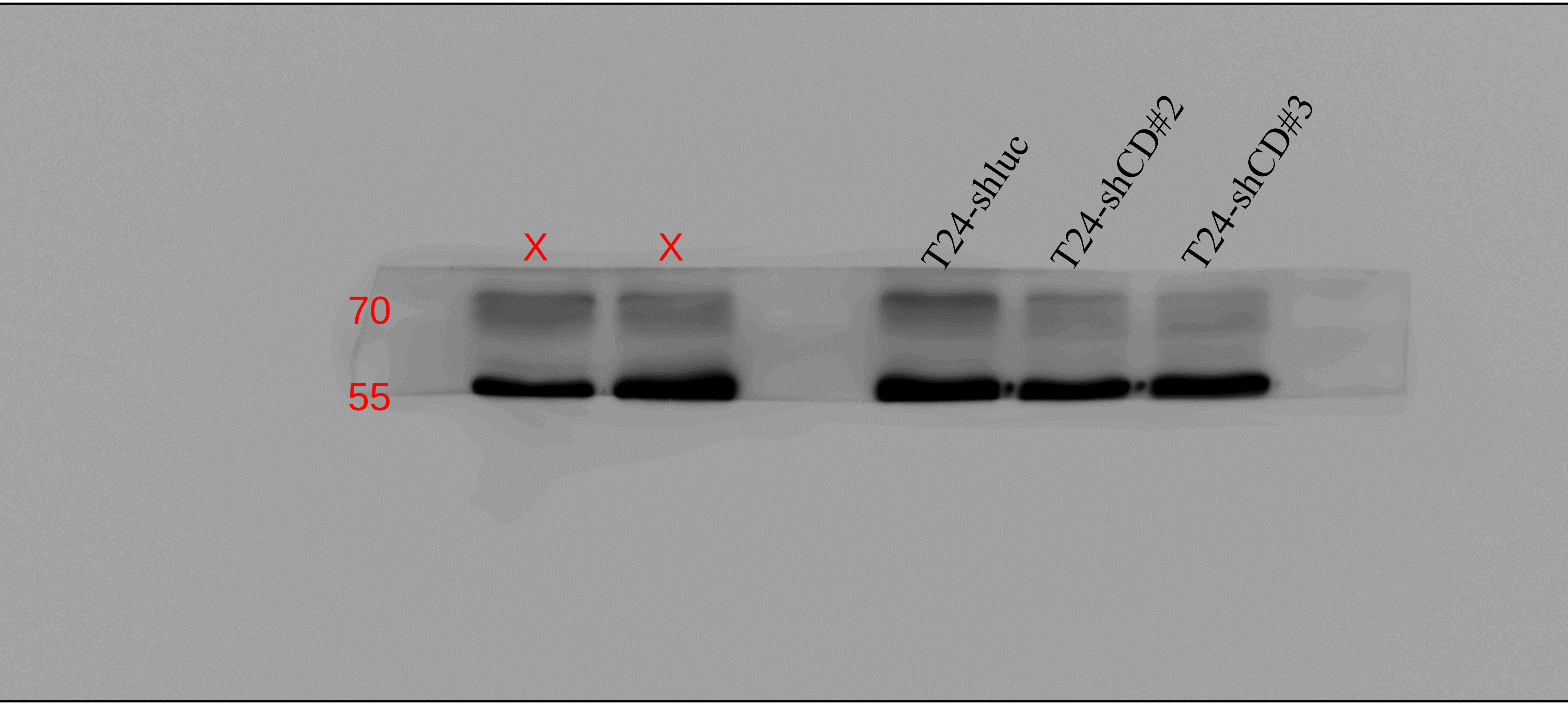

Fig. 3E anti-β-actin

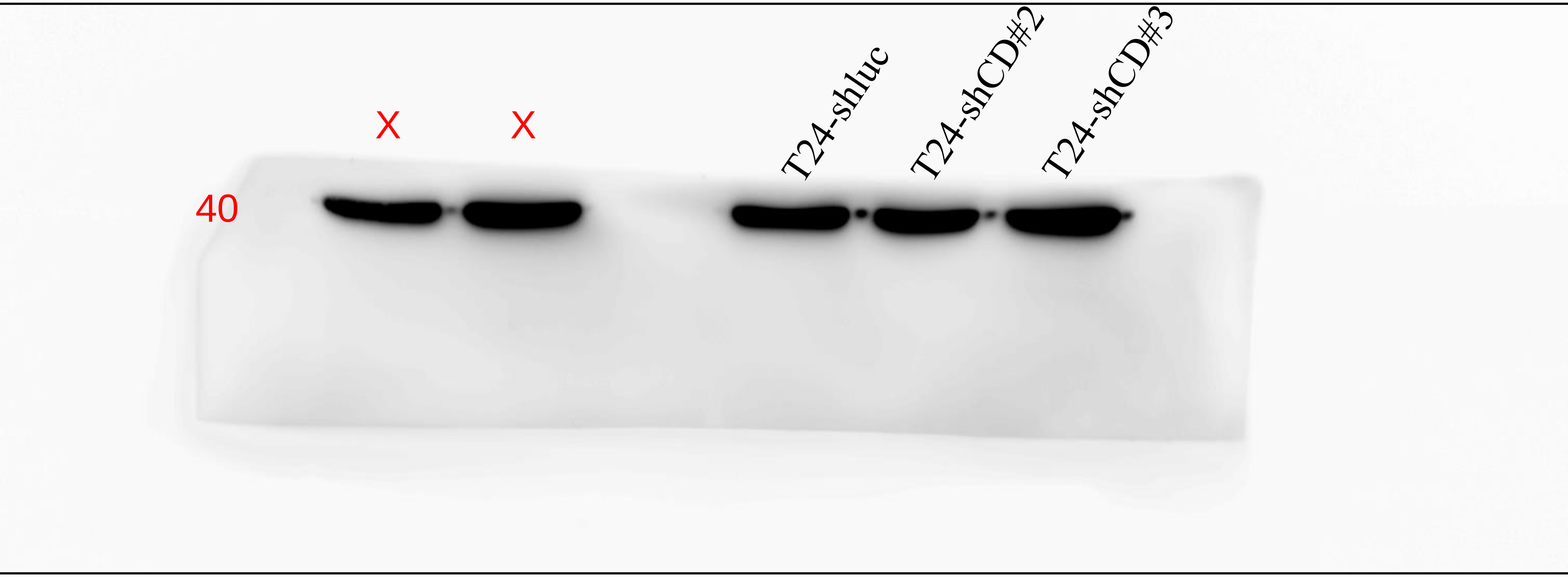

Fig. 4A anti-CDCP1

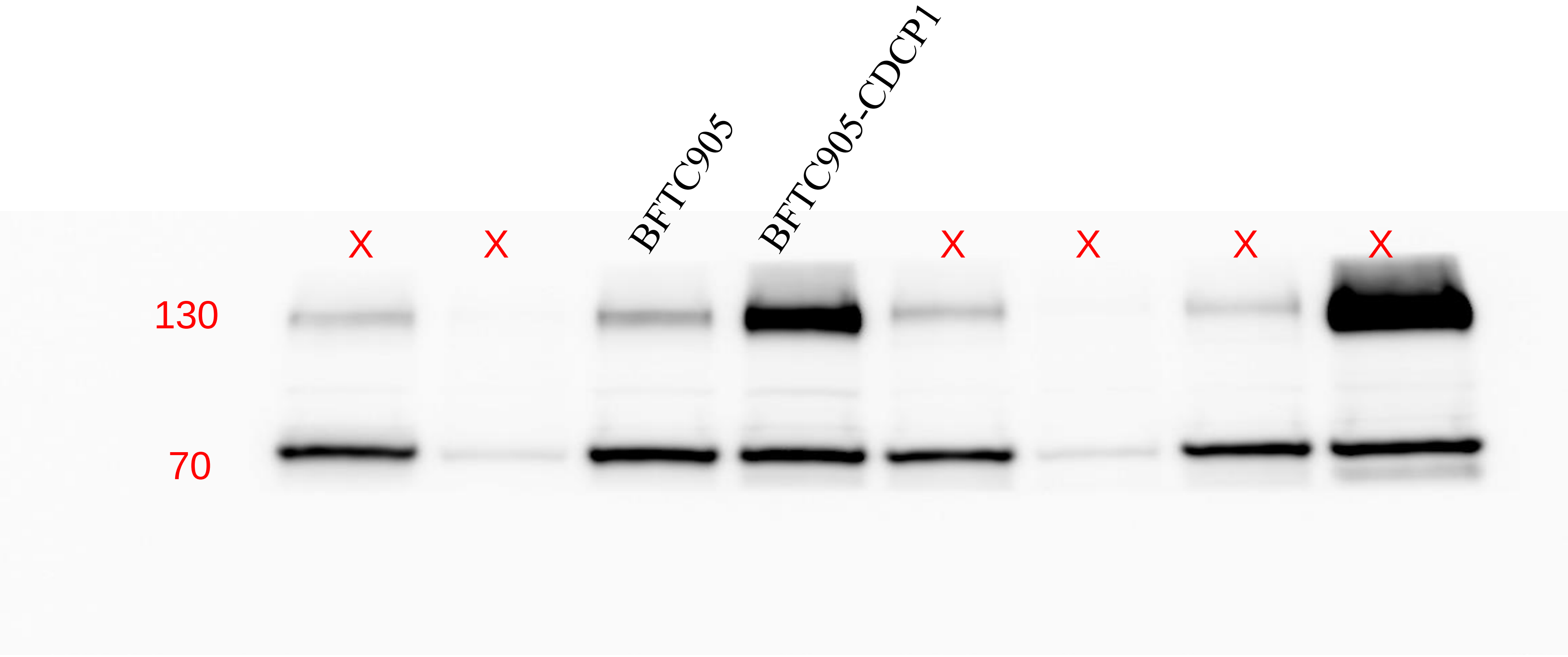

Fig. 4A anti-p-Src

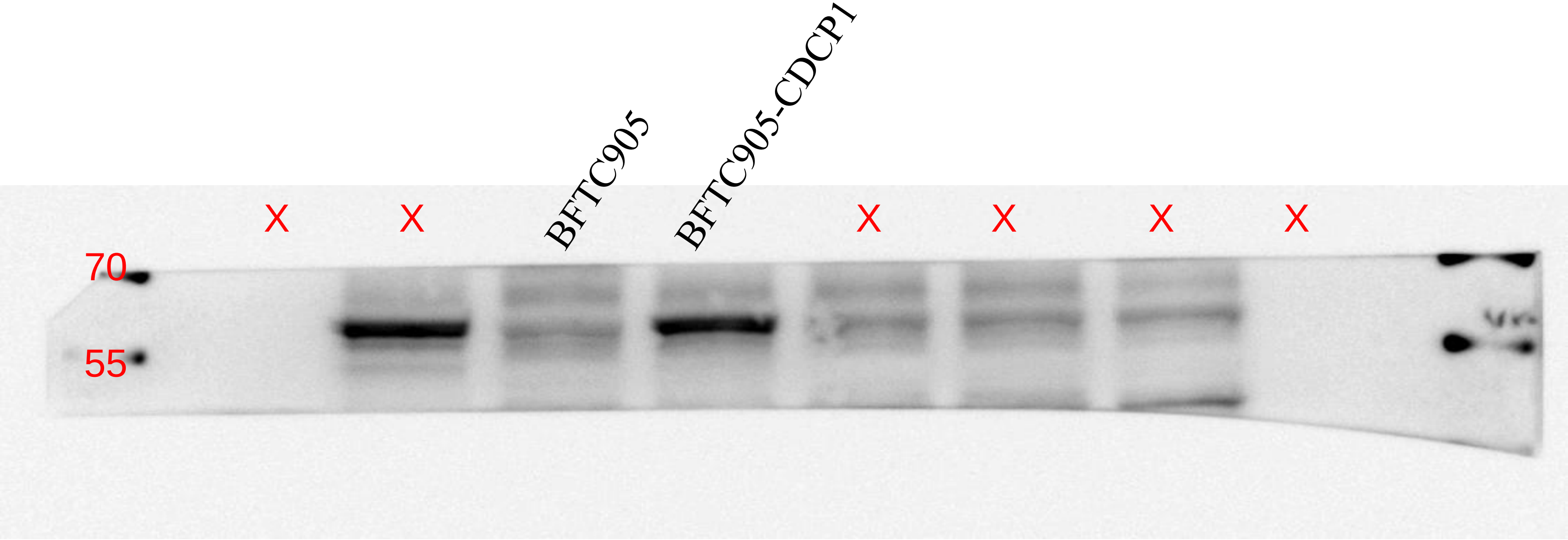

Fig. 4A anti-Src

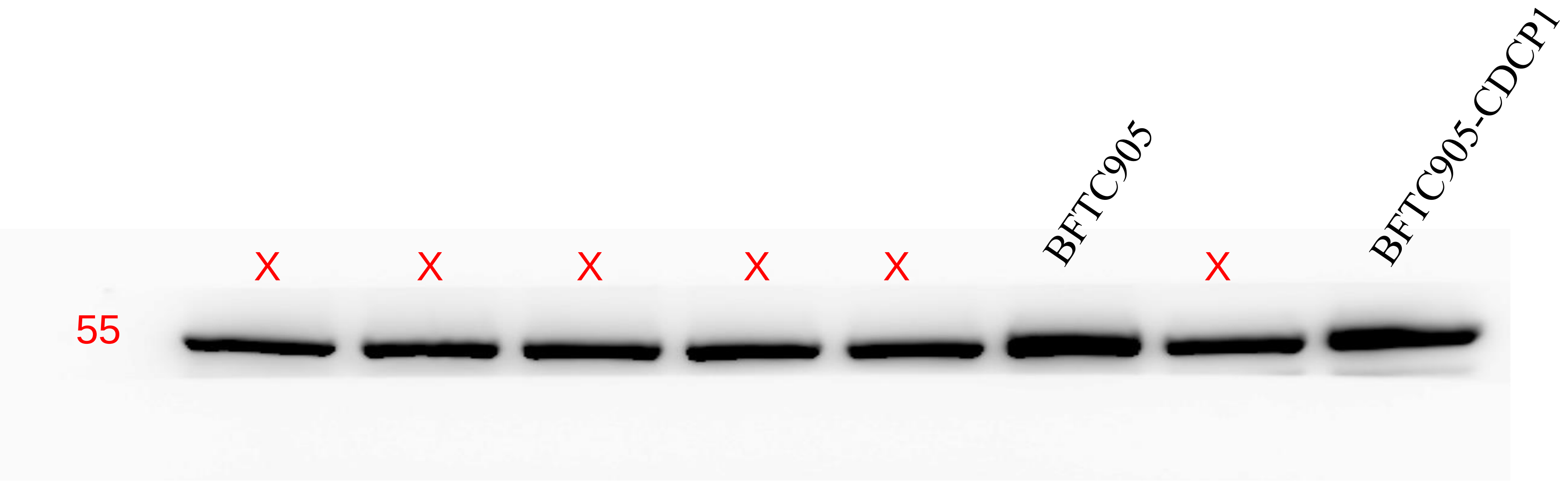

Fig. 4A anti-p-PKCδ

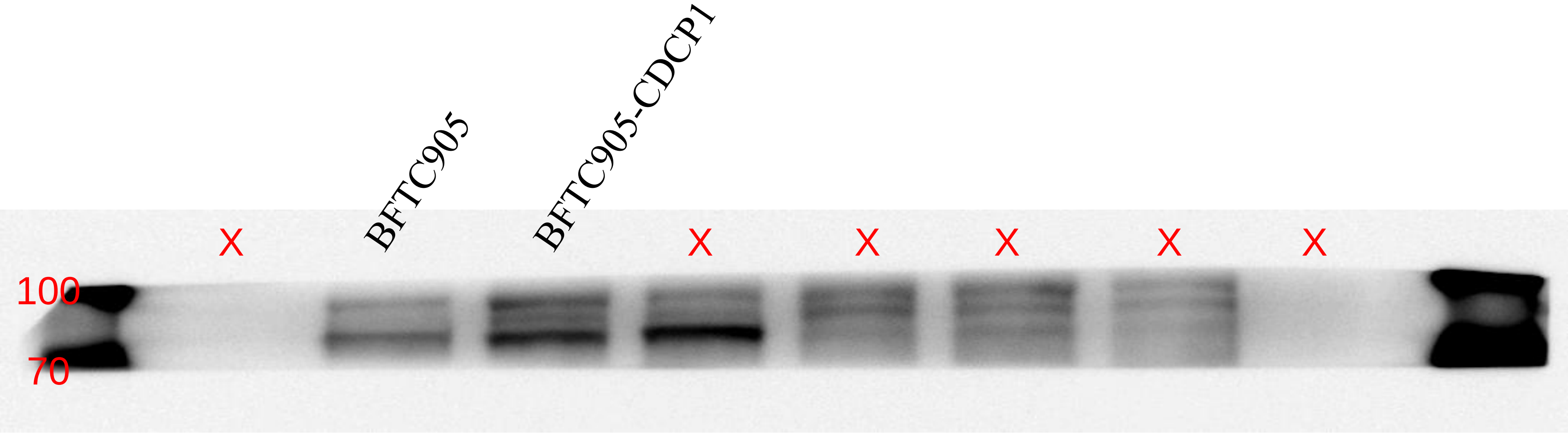

Fig. 4A anti-PKCδ

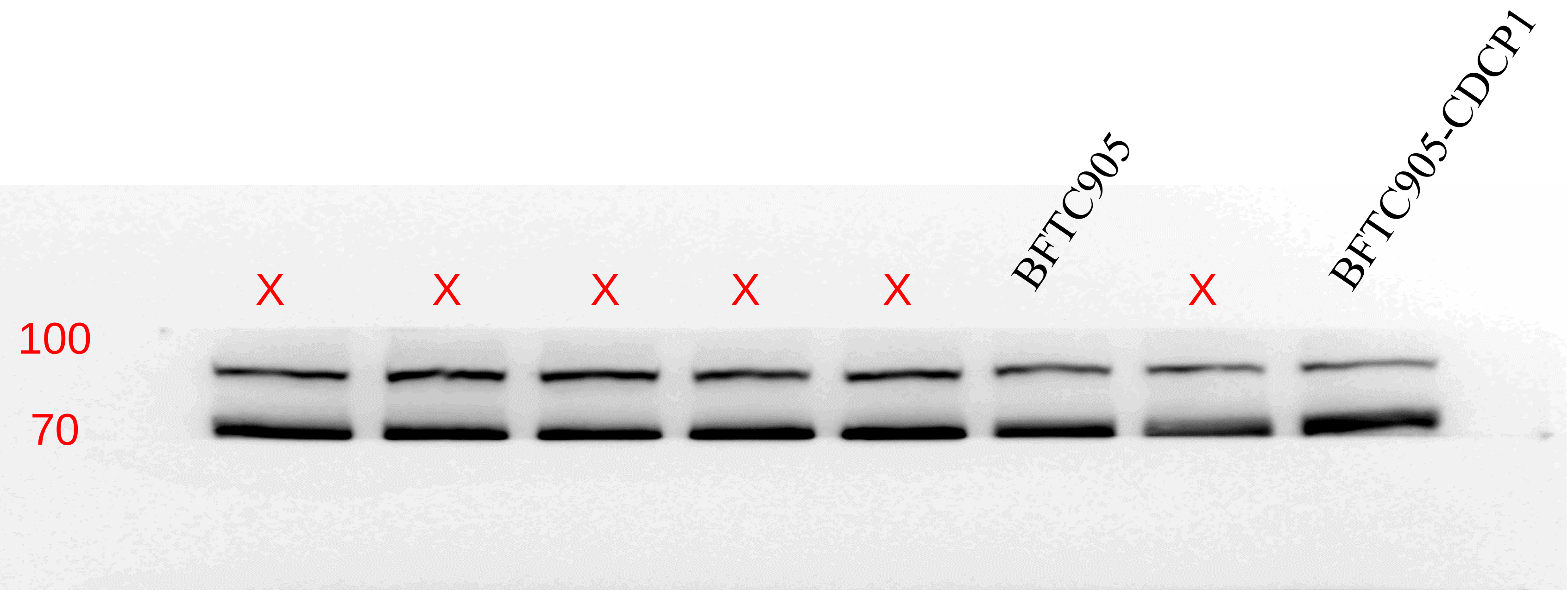

Fig. 4A anti-β-actin

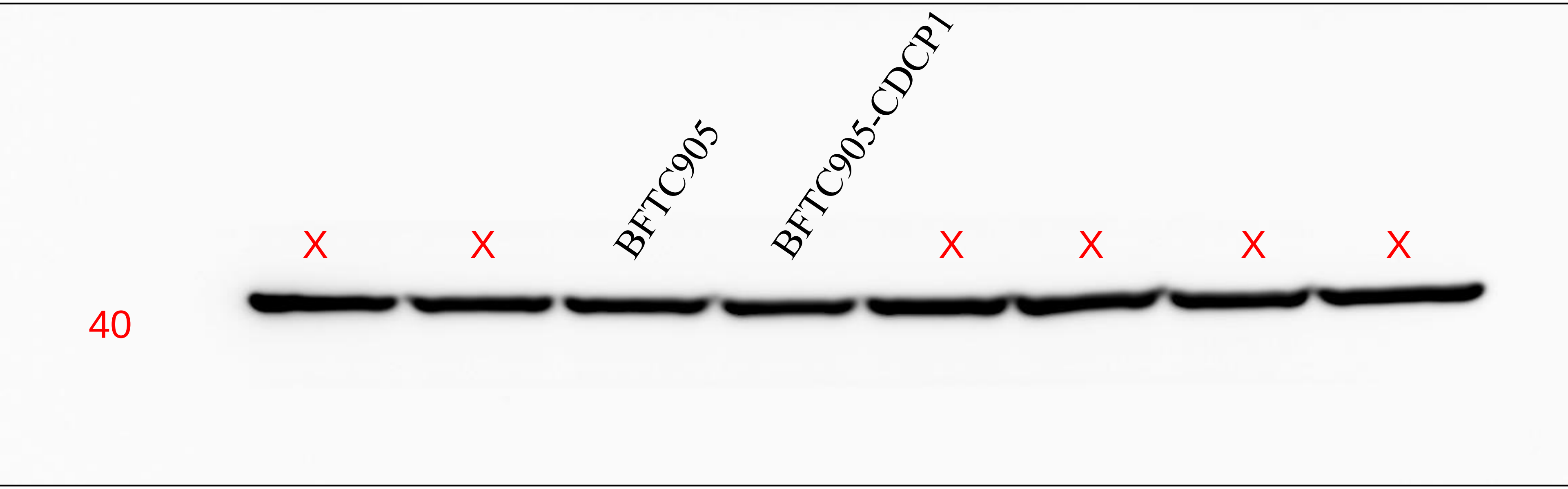

Supplement: S1 Raw images — (PDF) [file pone.0281873.s001.pdf]
