# Supplementary figures and images for: CDCP1 (CUB domain containing protein 1) is a potential urine-based biomarker in the diagnosis of low-grade urothelial carcinoma
Source: PLoS One. 2023 Mar 2;18(3):e0281873. doi: 10.1371/journal.pone.0281873 (PMC9980759; doi:10.1371/journal.pone.0281873)

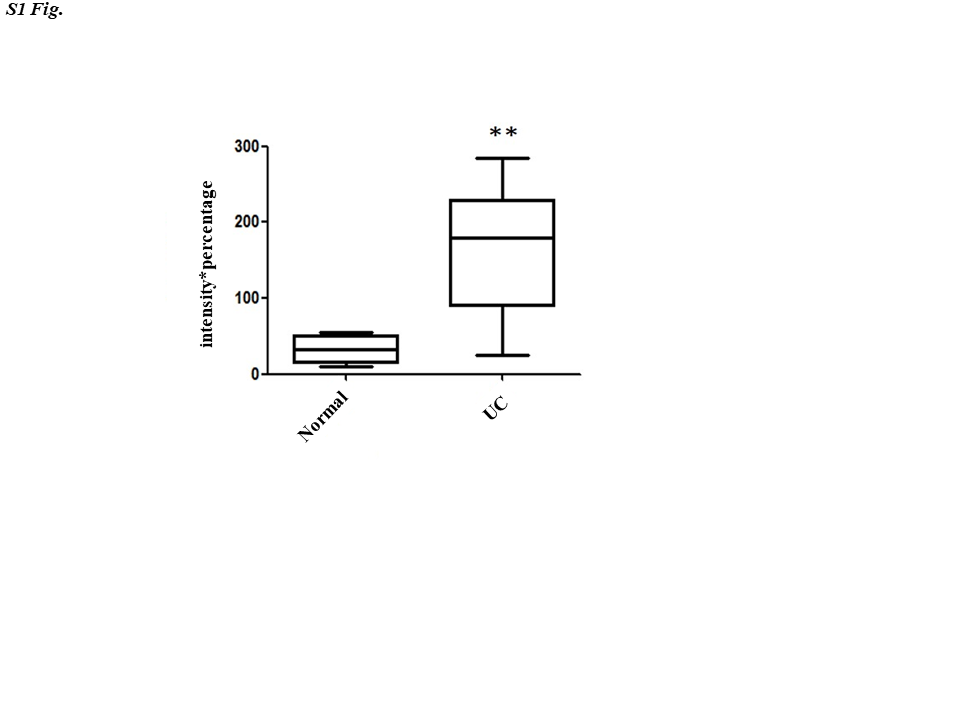

Supplement: S1 Fig — The product of the intensity and staining percentage of CDCP1 in specimens of UC patients was higher than that in specimens of normal persons. Statistical analyses were evaluated by two-tailed Student’s t-test. The results were presented as mean ± SD (normal n = 16; UC n = 133, *p < 0.05, **p < 0.01, ***p < 0.001). (TIF) [file pone.0281873.s003.tif]

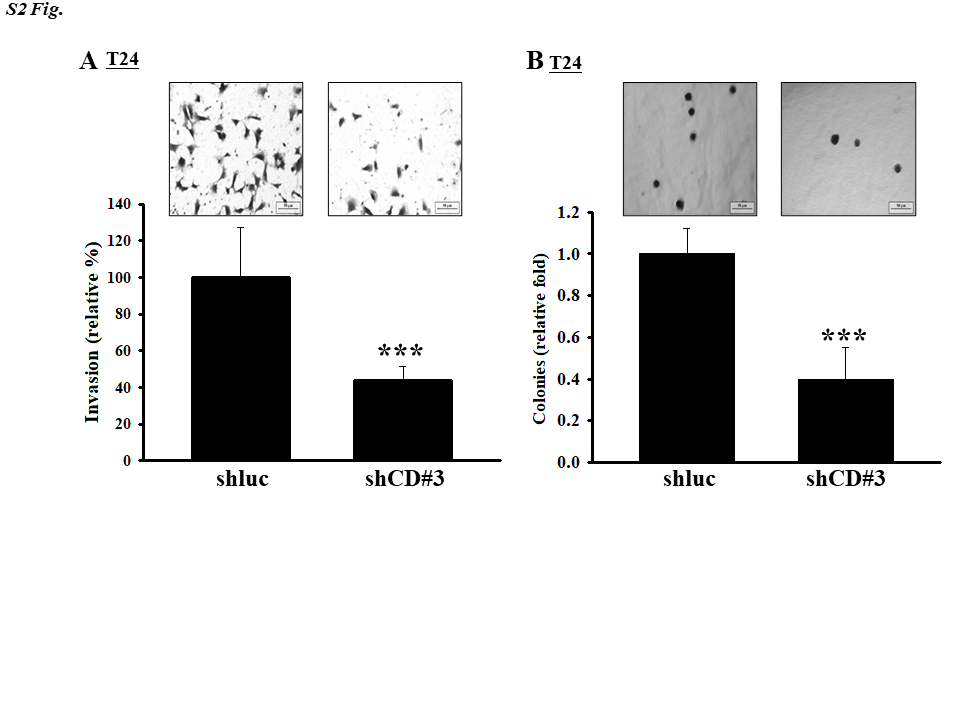

Supplement: S2 Fig — T24 cells were transfected the CDCP1 specific shRNA plasmids for 24 hrs, (A) The invasion abilities of CDCP1-silenced cells and parental cells were measured. (B) Colony formation in soft agar was also performed to compare parental and CDCP1-silenced cells as described in “Materials and methods” section. Statistical analyses were analyzed by unpaired two-tailed Student’s t-test. The results were presented as mean ± SD (n = 3, *p < 0.05, **p < 0.01, ***p < 0.001). (TIF) [file pone.0281873.s004.tif]
